# Margined Horn-Shaped Air Chamber for Body-Conduction Microphone

**DOI:** 10.3390/s23094565

**Published:** 2023-05-08

**Authors:** Shun Muramatsu, Yuki Kohata, Emi Hira, Yasuyuki Momoi, Michitaka Yamamoto, Seiichi Takamatsu, Toshihiro Itoh

**Affiliations:** 1 Department of Precision Engineering, Graduate School of Engineering, The University of Tokyo, Tokyo 113-8656, Japan; 2 Department of Precision Engineering, Faculty of Engineering, The University of Tokyo, Tokyo 113-8656, Japan; 3 Department of Veterinary Medical Sciences, Graduate School of Agricultural and Life Sciences, The University of Tokyo, Tokyo 113-8657, Japan

**Keywords:** wearable device, collar, scratching sound, body-conducted sound, air chamber

## Abstract

The sound amplification ratios of sealed air chambers with different shapes were quantitatively compared to design a body-conduction microphone to measure animal scratching sounds. Recently, quantitative monitoring of scratching intensity in dogs has been required. We have already developed a collar with a body-conduction microphone to measure body-conducted scratching sounds. However, the air chamber, one of the components of the body-conduction microphone, has not been appropriately designed. This study compared the amplification ratios of air chambers with different shapes through numerical analysis and experiments. According to the results, the horn-shaped air chamber achieved the highest amplification performance, at least for sound frequencies below 3 kHz. The simulated amplification ratio of the horn-shaped air chamber with a 1 mm height and a 15 mm diameter was 52.5 dB. The deformation of the bottom of the air chamber affected the amplification ratio. Adjusting the margin of the margined horn shape could maintain its amplification ratio at any pressing force. The simulated and experimental amplification ratios of the margined horn-shaped air chamber were 53.4 dB and 19.4 dB, respectively.

## 1. Introduction

Recently, the demand for dogs as companion animals has increased to relieve mental distress caused by measures against COVID-19 [1], and medical technology for dogs has developed remarkably. Conversely, for dermatosis, which has the highest prevalence among canine diseases, the evaluation of the severity and efficacy of treatment still depends on the subjective judgment of veterinarians and owners [2,3,4]. However, such judgments do not always lead to appropriate evaluations [5]. Thus, an objective method of evaluation is required. The severity of pruritic dermatoses, such as allergic dermatitis, is strongly associated with scratching behavior [6,7]. Therefore, objective and quantitative monitoring of the frequency and intensity of scratching behavior is a valuable technique for veterinary medicine.

Previous studies have attached an accelerometer to a dog’s collar and estimated the scratching behavior using statistics [3,8,9] or machine learning [10,11,12,13]. They can estimate the frequency of scratching; however, they cannot estimate the scratching intensity using scratching acceleration because scratching acceleration does not indicate scratching vibration but a body motion caused by scratching. Conversely, some studies have detected scratching intensity by measuring body-conducted sounds using a mechano-acoustic sensor adhered to human skin [14,15]. However, these methods cannot be applied to dogs because a dog’s hair interrupts the attachment of a mechano-acoustic sensor.

**Figure 1:**
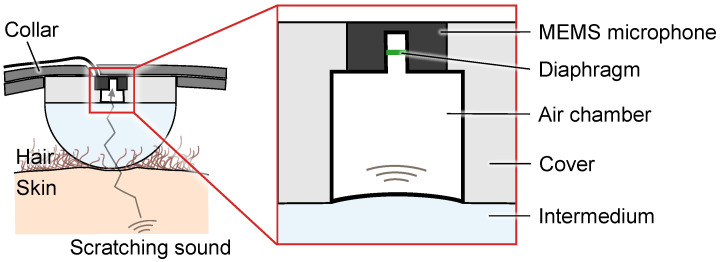
Our proposed body-conduction microphone for sensing scratching sounds.

Therefore, we aim to develop a scratching intensity monitoring system for animals. In this system, a body-conduction microphone is embedded in the dog’s collar and measures body-conducted scratching sounds. The scratching intensity is estimated using the measured sounds. We have already developed a wearable scratching sound-sensing device with body-conduction microphones and demonstrated scratching sound measurements for an actual dog [16]. Figure 1 depicts the proposed body-conduction microphone. This microphone consisted of an intermedium and a sealed air chamber. The intermedium can transmit body-conducted scratching sounds from the dog’s skin to the air chamber via acoustic impedance matching. These sounds transmit the boundary between the intermedium and the dog’s body surface via transmittance *T*, calculated as follows:

(1) $$T=1-{\left(\frac{Z_{a}-Z_{b}}{Z_{a}+Z_{b}}\right)}^{2}.$$

Here, *Z*_a_ and *Z*_b_ denote the characteristic acoustic impedance of the intermedium and the dog’s body, respectively. By selecting the material of the intermedium with acoustic impedance close to the body surface and away from air, it is possible to attenuate airborne sounds while transmitting body-conducted sounds effectively. The sensitivity of the body-conduction microphone prototype to body-conducted sounds relative to the sensitivity of airborne sounds has been calculated as 46 dB [16]. This indicates that general ambient sounds appearing in the surroundings at home are mostly eliminated by the effect of the intermedium. Conversely, the air chamber can amplify only body-conducted sounds using an instrument such as a stethoscope [17]. Specifically, body-conducted sounds reaching the upper surface of the intermedium cause the lower surface of the air chamber to vibrate. This vibration changes the air pressure over a large area, and this pressure vibration is propagated as a considerably amplified sound pressure to the small diaphragm of the MEMS microphone above the air chamber.

However, the air chamber has not yet been appropriately designed. Our body-conduction microphone needs to measure small scratching sounds. However, simple electrical amplification also amplifies airborne sounds and electrical noise. Therefore, the air chamber should have a higher amplification ratio to amplify only body-conducted sounds. In other words, the shape of the air chamber with a higher amplification ratio should be clarified. Generally, a bell-shaped air chamber is used for stethoscopes to amplify sounds [17]. A cylindrical air chamber was adopted in one study [18]. In another study, a trumpet bell shape and a shallow bell shape were adopted for the air chamber, and it was reported that the trumpet bell shape achieved a higher amplification ratio than the shallow bell shape [19]. However, there has been no quantitative comparison of the amplification ratios of air chambers with these four shapes. Therefore, we should compare the amplification ratios of these shaped air chambers. In addition, when our body-conduction microphone comes into contact with any object, including the dog’s skin, the intermedium is deformed because of its softness. This deformation makes the bottom of the air chamber deform in an arc and may change the amplification performance. This phenomenon does not occur in stethoscopes and has not yet been discussed. In this study, we aim to compare the amplification ratios of air chambers with different shapes, considering the deformation of the intermedium, and determine a suitable air chamber shape for our body-conduction microphone.

## 2. Materials and Methods

### 2.1. Simulation Methods

#### 2.1.1. Geometric Parameters

**Figure 2:**
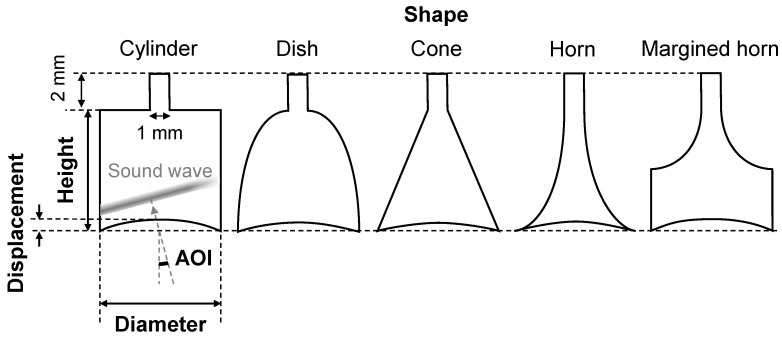
Five geometric parameters of the air chamber for the simulation.

Figure 2 shows five geometric parameters: shape, height, diameter, displacement, and angle of incidence (AOI). First, five shapes, namely, cylinder, dish, cone, horn, and margined horn, are considered. The cylinder is the simplest shape, and the air chamber of our body-conduction microphone has this shape. The cone corresponds to the bell shape mentioned in the introduction. The dish and horn correspond respectively to the shallow bell and trumpet bell mentioned in the introduction. In addition, a margined horn is proposed. This shape consists of a cylindrical margin and a horn. The margin eliminates the effect of the deformation of the intermedium. The horn is adopted because it has a better amplification ratio than the dish [19]. Second, the height is swept from 1 mm to 5 mm to fit the thickness of the collar. Third, the diameter is swept from 1 mm to 15 mm to fit in the width of the collar. Fourth, the deformation is defined using displacement, as depicted in Figure 2. The displacement is swept from 0 mm to 1 mm because the lowest height in the simulation is 1 mm. The amount of displacement relative to the pressing force depends on the shape of the air chamber. Therefore, the relationship between the pressing force and the displacement was calculated through solid mechanics simulations using simulation software (COMSOL Multiphysics, COMSOL Inc., Stockholm, Sweden). The Young’s modulus of the intermedium, which we measured experimentally, was 72 kPa. Fifth, the AOI of scratching sounds also needs to be considered. Scratching sounds generated at the scratching point, where the skin and finger come into contact, propagate to the intermedium and are transmitted into the air chamber. The speed of sound in the silicone rubber (the material of the intermedium) is approximately 1000 m/s [20], and in air it is 340 m/s. This difference makes the AOI in the air chamber smaller than that in the intermedium. Specifically, the AOI is limited up to 19° (≈tan^−1^(1000/340)) in the air chamber. Therefore, we consider the AOI of the sound source to be from 0° to 20° in the simulation. The above parameters and their sweep ranges are described in Table 1.

#### 2.1.2. Conditions of Simulation

The propagation of scratching sounds in the air chamber is simulated via two-dimensional numerical analysis using the finite-difference time-domain (FDTD) method [21,22] written in Python. The area sound source is located at the bottom of the air chamber. The receiving point is located at the diaphragm of the MEMS microphone, considering the structure of the actual body-conduction microphone. The other conditions of the FDTD method are listed in Table 2.

#### 2.1.3. Signal Processing

The performance of each air chamber is evaluated using the amplification ratio, which is calculated as follows. First, the time-domain impulse response is observed using the FDTD method. The sampling frequency is 4 MHz and the time length is 1 ms. The impulse response is transformed into a frequency-domain power spectrum by fast Fourier transform. The mean power from 0 Hz to 3000 Hz is then calculated because the main frequency of the scratching sound is lower than approximately 2000 Hz [15,23]. Finally, the amplification ratio is calculated as follows:

(2) $$r_{\mathrm{Sim}}=10\log{\left(\frac{p}{p_{\mathrm{Ref}}}\right)}^{2}.$$

Here, *r*_Sim_ denotes the amplification ratio and *p* denotes the mean power. *p*_Ref_ denotes a reference power and corresponds to the mean power of the cylinder shape with a height of 1 mm and a diameter of 1 mm. This shape is regarded as a case without the air chamber. The amplification ratio is squared to adjust the result of the two-dimensional simulation to that of a three-dimensional (3D) one.

The air chamber modifies the frequency response of the microphone due to resonance [23], and the resonant frequency depends on the size of the air chamber. For our application, the wavelength of the target scratching sound is longer than 170 mm because its frequency is lower than 2000 Hz [15,24]. Because this wavelength is sufficiently long compared with the structure of the air chamber (several millimeters), resonance effects can be ignored for our air chamber. Therefore, the frequency response is not discussed in this study.

### 2.2. Experimental Methods

A body-conduction microphone with an air chamber with the same characteristics as in the simulation is fabricated and evaluated with the experimental amplification ratio. The body-conduction microphone consists of a MEMS microphone (ICS-40180, InvenSense Inc., San Jose, CA, USA) installed on a MEMS microphone board (BOB-18011, SparkFun Electronics, Niwot, CO, USA), an intermedium, and an air chamber. Because this study simply needs to compare the amplification ratios of different air chambers, the microphone performance is not particularly important. Conversely, in practical use, three characteristics are important. The first is to have an appropriate frequency range for the scratching sound. The second is to have an analog output because the measured sounds need to be filtered before analog-to-digital conversion for anti-aliasing. The third is to have a high acoustic overload point (AOP). A low AOP distorts scratching sounds because loud body motion noise can easily saturate the signal. The reason for selecting the microphone used in this study is that it has a suitably low cut-off frequency, an analog output, and a high AOP. The original intermedium used in our body-conduction microphone is hemispherical. However, for correct evaluation, it was made cylindrical so that the amount of incoming sound would not change depending on the contact method in this experiment. The cylindrical intermedium (20 mm diameter, 10 mm height) was made of silicone rubber (KE-1308, Shin-Etsu Chemical Co., Ltd., Tokyo, Japan). The mold for forming the silicone rubber was made of polycarbonate using a 3D printer (Raise 3D E2, Raise 3D Technologies, Inc., Irvine, CA, USA). The cover for generating the air chamber was made of poly-lactic acid (PLA) and was also produced using the 3D printer. The stacking pitch was 0.01 mm. These three components were taped together with double-sided tape, with a hole created for the microphone so that the air chamber and the microphone hole would be air coupled. In addition, an adhesive was used to reinforce its joints from the outside, sealing the air chamber.

**Figure 3:**
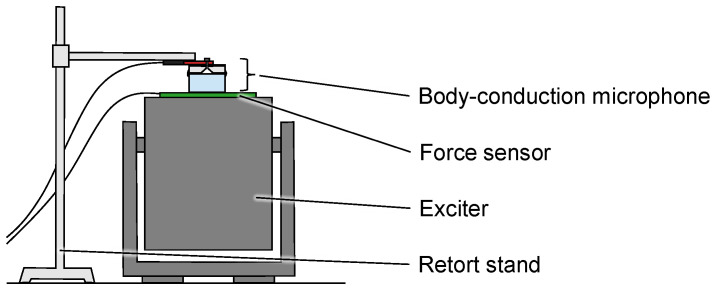
The setup of the experiment.

Figure 3 shows the experiment setup. The sine wave is output from the exciter (PM-100, Dynalabs, Ankara, Turkey). A single-frequency sine wave is used in the experiment, allowing for noise removal by filtering. The frequency is set to 300 Hz, this being within the range of scratching sounds. The fabricated body-conduction microphone measures the vibration in contact. A force sensor (FSR406, Interlink Electronics Inc., Camarillo, CA, USA) is inserted between the exciter and the body-conduction microphone to measure the pressing force. The measured sound is logged using a datalogger (DR-7100, Ono Sokki Co., Ltd., Yokohama, Japan) with a sampling frequency of 48 kHz and 16-bit resolution. For each body-conduction microphone, 30 s of sound data are measured. The data are filtered to extract 300 Hz using a band-pass filter (Butterworth, 5th order). The mean square value of the measured signal is calculated. The amplification ratio is then calculated as follows:

(3) $$r_{\mathrm{Exp}}=10\log\frac{p^{\prime }}{{p_{\mathrm{Ref}}}^{\prime }}.$$

Here, *r*_Exp_ denotes the amplification ratio and *p*′ denotes the mean square value. *p*_Ref_ denotes a reference mean square value and corresponds to the value of the cylinder shape with a height of 1 mm and a diameter of 1 mm (the same as in the simulation).

## 3. Results

### 3.1. Simulation Results

#### 3.1.1. Influence of Shape, Height, and Diameter

**Figure 4:**
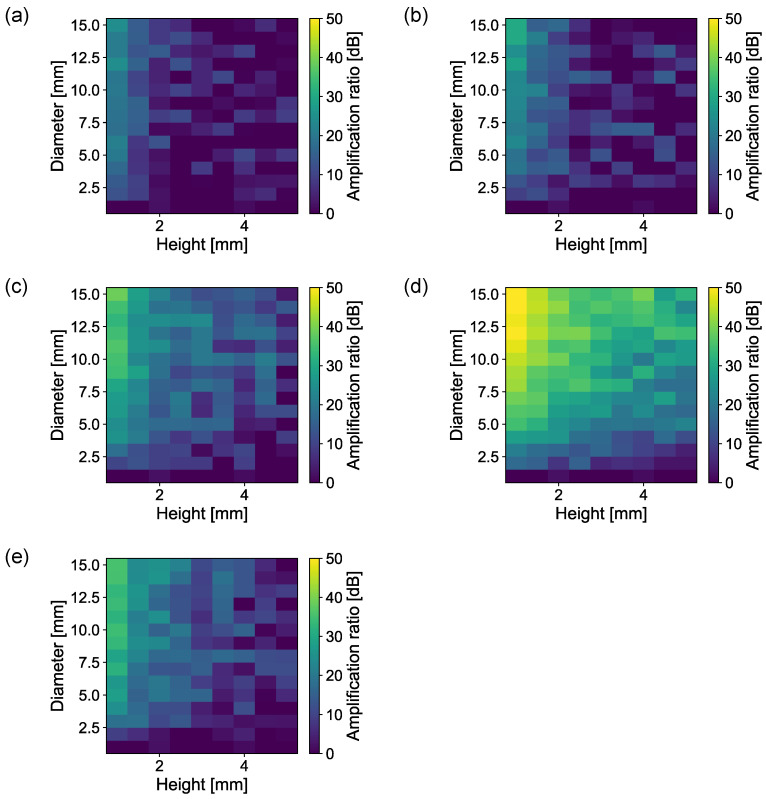
Amplification ratios against diameter and height. (**a**) Cylinder. (**b**) Dish. (**c**) Cone. (**d**) Horn. (**e**) Margined horn.

Figure 4 shows the amplification ratios of the cylinder (a), dish (b), cone (c), horn (d), and margined horn (e) shapes with the swept heights and diameters. The displacement and AOI are 0.0 mm and 0°, respectively. The lower height and higher diameter achieve higher amplification ratios for all shapes. The horn shape achieves the highest amplification ratio among the five shapes. In particular, with a height of 1 mm and a diameter of 15 mm, it has the best performance. Conversely, the cylinder and dish shapes achieve the lowest amplification ratios. These results indicate that the diameter-to-volume ratio significantly affects the amplification ratio.

**Figure 5:**
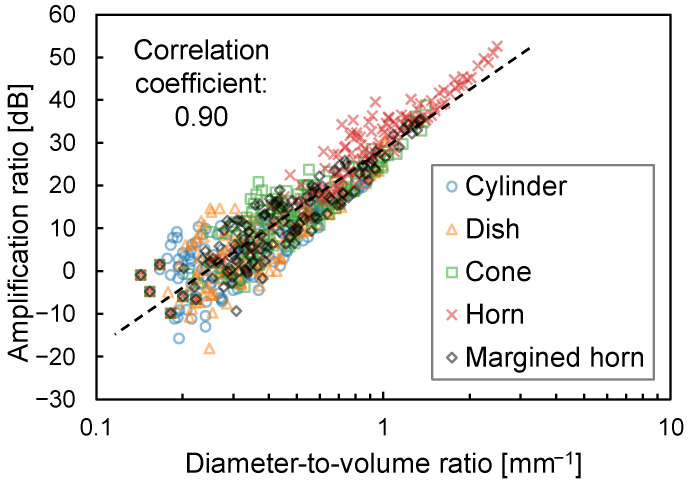
Relationship between amplification and diameter-to-volume ratios. The dashed line indicates the regression line.

Figure 5 shows the amplification ratios against the diameter-to-volume ratios. The dashed line represents the regression line, and the correlation coefficient is 0.90. This indicates that the amplification ratio is strongly correlated with the diameter-to-volume ratio. Conversely, the distribution has a longitudinal width. In other words, the amplification ratio is volatile even at the same diameter-to-volume ratio.

**Figure 6:**
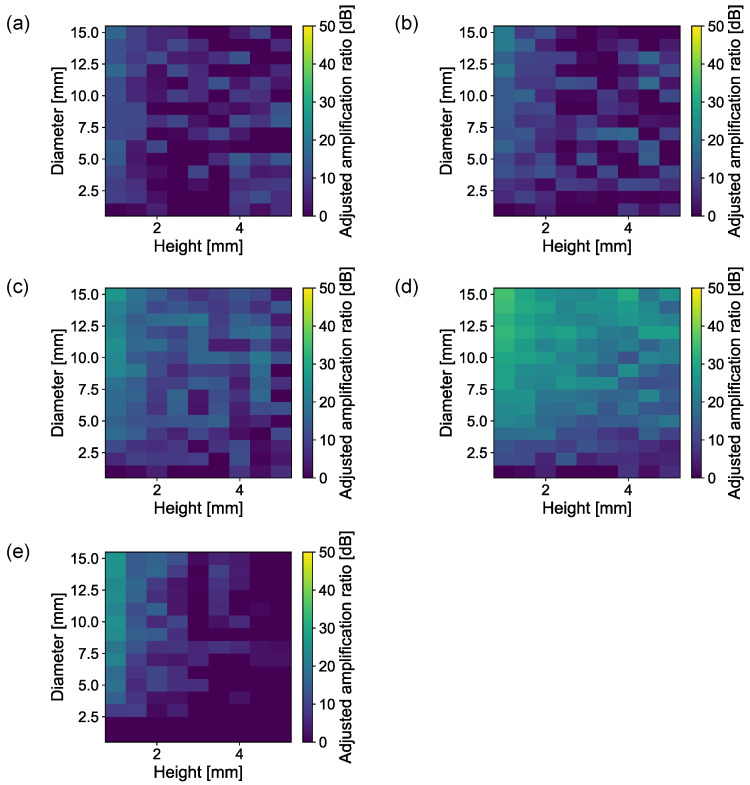
Adjusted amplification ratio against diameter and height. (**a**) Cylinder. (**b**) Dish. (**c**) Cone. (**d**) Horn. (**e**) Margined horn.

To investigate this volatility, the amplification ratio was adjusted to have the same diameter-to-volume ratio. Specifically, the amplification ratio was multiplied by the reciprocal of the diameter-to-volume ratio. Figure 6 depicts the adjusted amplification ratio. If only the diameter-to-volume ratio affects the amplification ratio, these five graphs can be considered to have the same characteristics. However, the horn shape has a higher amplification ratio than the other shapes, followed by the cone and margined horn shapes. This result indicates that the geometric characteristics of the air chamber shape affects the amplification ratio regardless of the diameter-to-volume ratio.

**Figure 7:**
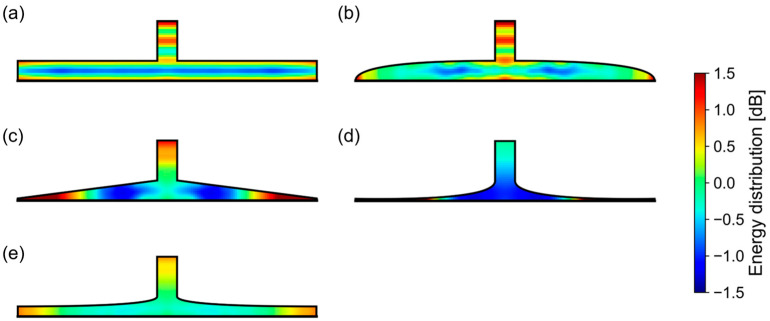
Energy distribution of sound in air chambers. (**a**) Cylinder. (**b**) Dish. (**c**) Cone. (**d**) Horn. (**e**) Margined horn.

To investigate how the geometric characteristics of the air chamber shape affect its amplification ratio, the energy distributions of the sounds in each air chamber were calculated, and these are shown in Figure 7. The height and diameter were then fixed at 1 mm and 15 mm, respectively. The energy distribution was calculated as a sum of the power for each element during the simulation. The energy distribution of 0.0 dB is defined as the mean of all areas in the air chamber. In this figure, the cylinder and dish shapes have striped patterns, indicating standing waves. We assume that a standing wave indicates that the energy of the sounds stagnates and does not flow into the microphone. For example, most of the sound propagated into the cylinder-shaped air chamber continued to reflect off the upper and lower surfaces of the chamber. Conversely, the other shapes do not have a standing wave. We believe that this is the reason why the horn shape has a higher amplification ratio than the others.

#### 3.1.2. Influence of Displacement

**Figure 8:**
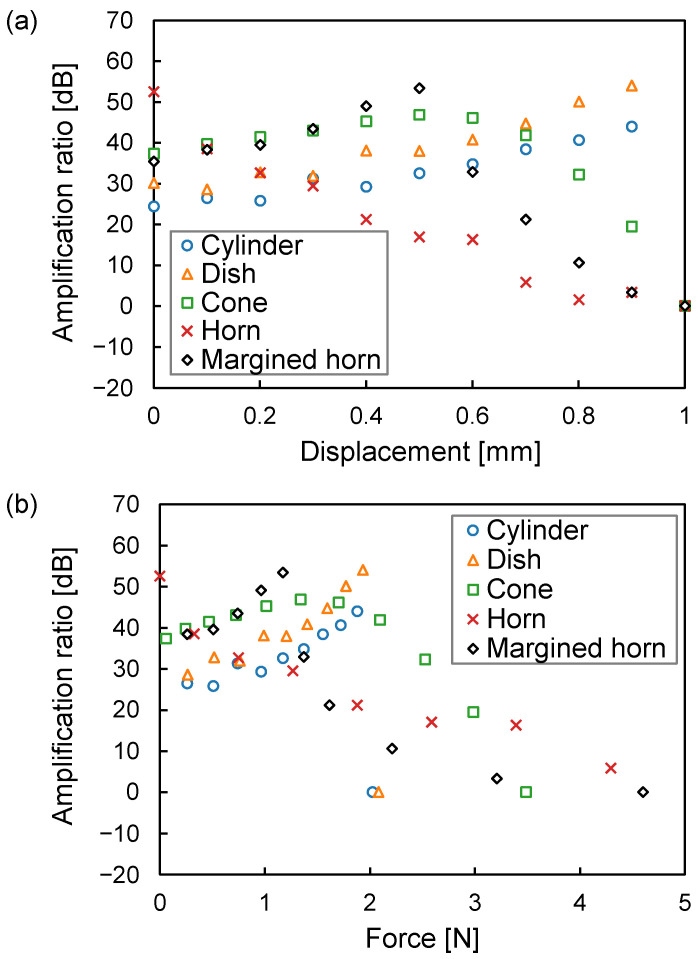
Relationship between the amplification ratio and the intermedium’s deformation. (**a**) Displacement. (**b**) Pressing force.

Figure 8a depicts the amplification ratio against displacement. The amplification ratio of the horn shape is 52.5 dB at 0 mm displacement and decreases sharply with an increase in displacement. This shows that space is vanishing from the outside as the intermedium deforms, suppressing the horn’s delicate skirt. As a result, its diameter is thought to shorten in effect. The amplification ratios of the cylinder and dish shapes increase with an increase in displacement. This indicates that, for these shapes, the deformation of the intermedium does not shorten its diameter in effect and simply reduces the air chamber volume. The amplification ratio of the margined horn shape increases up to a displacement of 0.5 mm, but decreases after that. This indicates that the influence of skirt squashing appears at displacements higher than 0.6 mm. In other words, the intermedium and the upper surface of the air chamber begin to come into contact with each other. For each shape, a displacement of 1 mm causes the intermedium to reach the microphone hole on the top of the air chamber. This indicates that the air chamber becomes a simple cylinder with a 1 mm width, and the amplification ratio is 0 dB at this time. The cone, horn, and margined horn shapes gradually approach 0 dB. Conversely, the cylinder and dish shapes suddenly fall to 0 dB because their intermedia come into contact with the microphone holes suddenly, not gradually from the outside. Figure 8b depicts the amplification ratio against the pressing force. It differs from Figure 8a because the force required to deform the intermedium differs depending on the chamber shape. It is indicated that the amplification ratio of the margined horn shape is the peak shifted to the right while maintaining the peak value of the amplification ratio of the horn shape. This is the effect of the margin.

**Figure 9:**
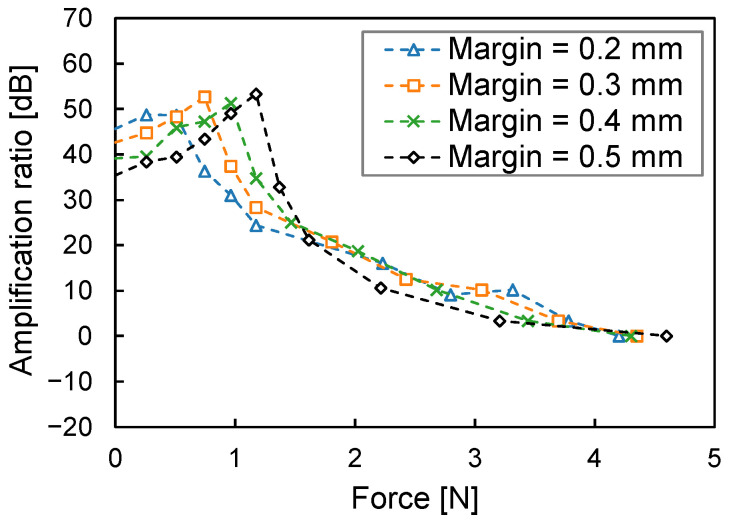
Relationship between the amplification ratio of the margined horn shape and the pressing force.

Although the margin has been fixed at half the height of the entire chamber for the margined horn shape, the peak can be shifted to any pressing force by adjusting the margin. For example, Figure 9 shows the relationship between the amplification ratio and the pressing force when the margin ranges from 0.2 mm to 0.5 mm and the whole height is 1 mm. The pressing force depends on the design of our final collar device. The current prototype device requires a pressing force of approximately 1 N, and a margin of 0.4 mm is appropriate and achieves a peak amplification ratio of 51.4 dB. In practice, we can design this margin simultaneously with the entire device.

#### 3.1.3. Influence of AOI

**Figure 10:**
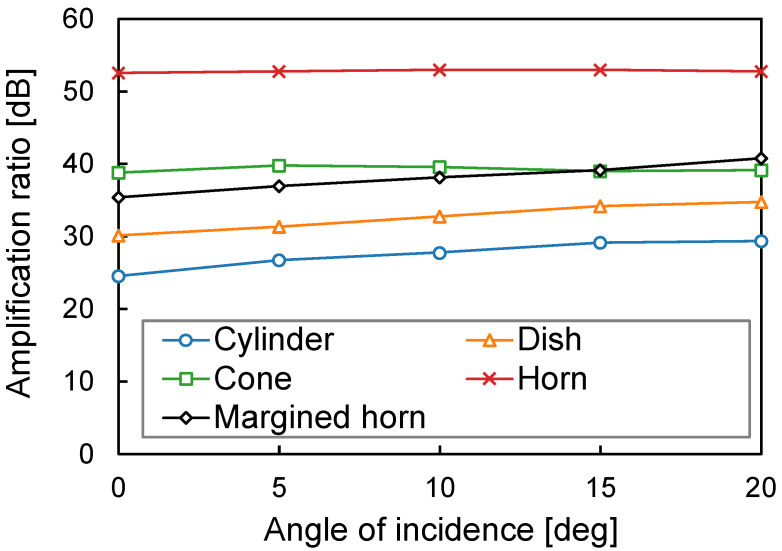
Relationship between the amplification ratio and the AOI.

Figure 10 shows the amplification ratios against the AOI. Although the cone and margined horn intersect, the others do not intersect each other. This indicates that the AOI is less influential than the shape and does not need to be considered in this study. Conversely, the amplification ratios increase slightly with the AOI, especially for the cylinder, dish, and margined horn shapes. We believe that a higher AOI reduces standing waves in the cylinder-shaped air chamber and increases the amplification ratio compared with a lower AOI. In summary, the body-conduction microphone can amplify sound wherever it occurs on a dog’s skin. This enhances the feasibility of our collar-based scratching sound sensing.

### 3.2. Experimental Results

**Figure 11:**
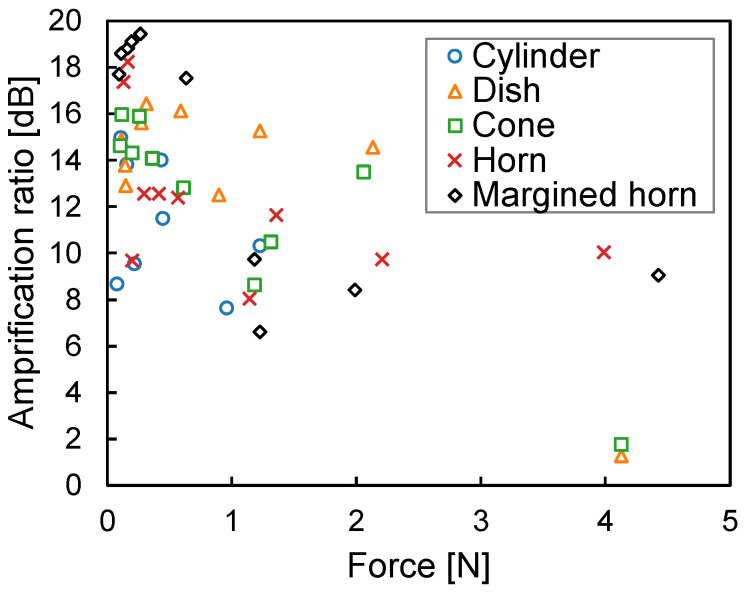
Relationship between the experimental amplification ratio and the pressing force.

The experiment evaluated the five shapes with a height of 1 mm and a diameter of 15 mm, these parameters having produced the highest amplification ratio in the simulation. The margin of the margined horn shape was 0.5 mm. Figure 11 shows the experimental amplification ratios against the pressing force of the five shapes. The margined horn achieved the highest amplification ratio among the five shapes, and its amplification ratio was 19.4 dB at a pressing force of 0.27 N. The simulated amplification ratio’s peak was 53.4 dB at 1.17 N, as depicted in Figure 8. The experimental amplification ratio was 34.0 dB lower than the simulated one. We believe that there are two reasons for this. The first is the fabrication method of the cover generating the air chamber. The cover was made using a fused deposition modeling-type 3D printer. This type may result in small gaps in the stacking of the material, and the air chamber may not have been completely sealed. The second is the characteristic acoustic impedance of the cover. Changing the material of the cover from PLA to a material with acoustic impedance farther from that of air can reduce the outflow of body-conducted sounds.

The pressing force providing the peak of the amplification ratio for the margined horn also deviates between the experiment and the simulation. We believe that there are two reasons for this. The first is that the material of the fabricated body-conduction microphone, including the glue, the double-sided tape, and the structure stacked by the 3D printer, absorb some of the force. The second is the surface roughness of the intermedium fabricated with silicone rubber. This roughness distorts the fine horn shape and causes an offset in displacement.

The amplification ratio of the margined horn appears to converge to approximately 9 dB instead of 0 dB. We believe that there are two reasons for this. The first is the fine patterns on the surface of the intermedium. Because the intermedium was formed by pouring in molds fabricated by the 3D printer, slight grooves due to stacking were patterned on the surface of the intermedium. These grooves prevent the sealing of the contact area between the intermedium and the microphone hole. The second is the inaccuracy of the pressing angle. In the experiment, the intermedium was pressed against the exciter using a retort stand. This method is not as accurate for pressing angles as simulation. The inclined pressing also prevents the sealing of the contact area between the intermedium and the microphone hole.

For the other shapes, the experimental results are consistent with the simulation, i.e., the amplification ratio decreases at a higher pressing force. Conversely, these plots deviate from the simulation at a lower pressing force. We believe that this is because of the offset mentioned above. In other words, assuming that 0 N in the experiment corresponds to approximately 1 N in the simulation, they are relatively consistent.

In summary, the experimental results are consistent with the simulations in that the margined horn has the highest performance and peaked distribution, even though there are differences between the experiment and the simulation.

## 4. Discussion

**Figure 12:**
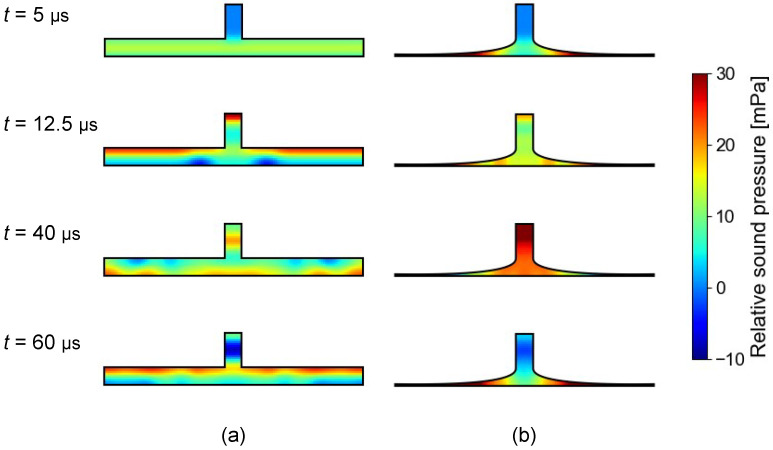
Transient sound propagation in air chambers. (**a**) Cylinder. (**b**) Horn.

There had previously been no quantitative comparison of the amplification ratios of air chambers with different shapes. This study shows that the horn-shaped air chamber achieves the highest amplification performance, at least for sound frequencies below 2 kHz. There are two reasons for this. The first is that the horn shape has a higher diameter-to-volume ratio, as is depicted in Figure 5. This is reasonable because of the following. The diameter corresponds to the amount of sound energy flowing into the air chamber. Conversely, the volume corresponds to the number of air molecules absorbing the sound energy. Therefore, the diameter-to-volume ratio corresponds to the energy used to vibrate certain air molecules. The second is that it is difficult for standing waves to exist within the horn shape, as is depicted in Figure 7. To explore this further, Figure 12 shows the transient sound propagation in the cylinder-shaped and horn-shaped air chambers. They are visualized as sound pressure distributions relative to the initial condition in which *t*, representing the time elapsed since the impulse wave was generated, is 5 μs, 12.5 μs, 40 μs, and 60 μs. The two shapes are adjusted to have the same diameter-to-volume ratio. In the cylinder-shaped chamber, high pressure is distributed on the upper surface of the chamber when *t* = 12.5 μs and 60 μs, and it is distributed on the lower surface when *t* = 40 μs. These results indicate that most of the sound continues to reflect off the upper and lower surfaces of the chamber and is not flowing into the microphone. Conversely, in the horn-shaped chamber, high pressure is distributed on both sides of the air chamber when *t* = 5 μs and 60 μs, and it is distributed near the center of the chamber, including the microphone, when *t* = 40 μs. These results indicate that most of the sound circulates well in the air chamber and is not stagnant. In summary, a horn-shaped air chamber should be adopted when fabricating a body-conduction microphone, including a stethoscope with a higher amplification ratio.

This study shows that the deformation of the bottom of the air chamber affects the amplification ratio, as is depicted in Figure 8. This issue is critical for our body-conduction microphone because the intermedium attached to the bottom of the air chamber is made from a very soft material. To address this issue, adjusting the margin can maintain the amplification ratio of the horn shape at any pressing force, as is depicted in Figure 11. We will monitor the pressing force when the prototype collar is attached to an actual dog to determine the appropriate margin.

We intend to develop a scratching intensity-monitoring system for animals using a collar with an embedded body-conduction microphone. The intermedium has been designed and evaluated [16]. Now, an air chamber has been designed in this study. The remaining issue is the selection of the MEMS microphone. Although we are using a capacitive MEMS microphone because of its low noise level and high sensitivity, we will attempt to use a piezoelectric MEMS microphones because they do not require a biasing voltage, have a wider dynamic range, and are more dustproof and waterproof than capacitive microphones [25]. Following this, we will complete the collar fabrication and move on to scratching intensity estimation. The fabricated collar device will be able to distinguish body-conducted sounds from ambient airborne sounds. However, it is difficult to distinguish scratching sounds from different body-conducted sounds generated by other behaviors. We will consider using an accelerometer embedded in the collar with a body-conduction microphone. There are some previous studies that have estimated scratching behavior using scratching acceleration measured by the collar [3,8,9,10,11,12,13]. We will utilize this approach and judge whether the measured body-conducted sound is a scratching sound using scratching acceleration.

## 5. Conclusions

The amplification ratios of air chambers with different shapes, considering the deformation of the intermedium, were quantitatively compared to design a body-conduction microphone to sense animal scratching sounds. The horn-shaped air chamber achieves the highest amplification performance, at least for sound frequencies below 3 kHz. The simulated amplification ratio of the horn-shaped air chamber with a height of 1 mm and a diameter of 15 mm was 52.5 dB. The deformation of the bottom of the air chamber affects the amplification ratio. Adjusting the margin of the margined horn shape can maintain its amplification ratio at any pressing force. The appropriate margin for practical use can be determined by monitoring the pressing force when the prototype collar is attached to an actual dog. The simulated and experimental amplification ratios of the margined horn-shaped air chamber were 53.4 dB and 19.4 dB, respectively. The experimental amplification ratio can be improved by improving the fabrication method of the cover. In future studies, we will complete the fabrication of the scratching sound-sensing collar based on these findings and move on to the next stage, i.e., actual dog scratching intensity estimation.

## Figures and Tables

**Table 1:** Sweep ranges of the parameters.

Parameter	Sweep Range
Shape	Cylinder, dish, cone, horn, and margined horn
Height (mm)	1–5
Diameter (mm)	1–15
Displacement (mm)	0–1
AOI (deg)	0–20

**Table 2:** Conditions of the FDTD method.

Variable	Value
Sound velocity (m/s)	3.405 × 10^2^
Atmosphere density (kg/m^3^)	1.242
Bulk modulus (Pa)	1.43954 × 10^5^
Space discrete width (m)	1.0 × 10^−4^
Time discrete width (s)	2.5 × 10^−8^
Analysis time (s)	1.0 × 10^−3^

## Data Availability

Not applicable.
